# Optimization of Mixed Numerology Profiles for 5G Wireless Communication Scenarios †

**DOI:** 10.3390/s21041494

**Published:** 2021-02-21

**Authors:** Noélia Correia, Faroq Al-Tam, Jonathan Rodriguez

**Affiliations:** 1 Center for Electronic, Optoelectronic and Telecommunications (CEOT), University of Algarve, 8005-139 Faro, Portugal; 2 Department of Computer Science, Aberystwyth University, Aberystwyth SY23 3FL, UK; faa14@aber.ac.uk; 3 Instituto de Telecomunicações, Universidade de Aveiro, 3810-193 Aveiro, Portugal; jonathan@av.it.pt

**Keywords:** 5th-Generation mobile networks, radio resource management, mixed numerology

## Abstract

The management of 5G resources is a demanding task, requiring proper planning of operating numerology indexes and spectrum allocation according to current traffic needs. In addition, any reconfigurations to adapt to the current traffic pattern should be minimized to reduce signaling overhead. In this article, the pre-planning of numerology profiles is proposed to address this problem, and a mathematical optimization model for their planning is developed. The idea is to explore requirements and impairments usually present in a given wireless communication scenario to build numerology profiles and then adopt one of the profiles according to the current users/traffic pattern. The model allows the optimization of mixed numerologies in future 5G systems under any wireless communication scenario, with specific service requirements and impairments, and under any traffic scenario. Results show that, depending on the granularity of the profiles, the proposed optimization model is able to provide satisfaction levels of 60–100%, whereas a non-optimized approach provides 40–65%, while minimizing the total number of numerology indexes in operation.

## 1. Introduction

Boosting the data rate has been the main objective of wireless communication systems over this last decade. Long-Term Evolution (LTE) aimed at improving user data rate experience, and all the enhancements were centered on this main requirement, while the needs of services like virtual reality and the Internet of Things (IoT) were not properly taken into account. Currently, we are experiencing the 5th-Generation (5G) mobile era, where the objective is to allow the coexistence of different services and heterogeneous users on the same networking platform, besides looking for improvements of the data rate.

5G is a multi-use-case environment designed to reach almost all life aspects, from industry and health care to online games and vehicle-to-everything (V2X) communications. The current 5G is, however, just the beginning of more advanced communication systems. The service stack will continue to grow, and new applications are expected to be developed. It is no surprise that new requirements will also emerge in the near future, related to energy in particular, and more advanced designs will undoubtedly be needed [1].

The current 5G specifications describe it as a multi-purpose communication ecosystem. That is, it will be an umbrella for several services where each service is associated with a bundle of requirements (see [2] for further details). In order to support them, 5G is equipped with new features. Among them, flexibility is the most important one [3]. Supporting multiple use-cases requires a flexible design not only on radio access methods and deployment options, but also on the frame structure and operations it supports. The corner stone to achieve such flexibility is the new design of the 5G new radio (NR).

The NR is built on four main pillars: (i) new spectrum; (ii) massive MIMO and beamforming; (iii) multi-connectivity; (iv) network flexibility and virtualization (numerology, slicing, NFV/SDN). The flexibility of NR spreads all over the system planes. Not only on the frame structure, but also on the operation and protocol stack logic. Furthermore, NR is user-centric, and bandwidth parts (BWPs) can be used to fit user equipment (UE) temporal requirements with dynamic transmission time interval (TTI) lengths and dynamic time division duplex (DTDD) [4]. In short, the 5G NR is designed with components that are flexible, ultra-lean, and forward-compatible [5].

**Figure 1:**
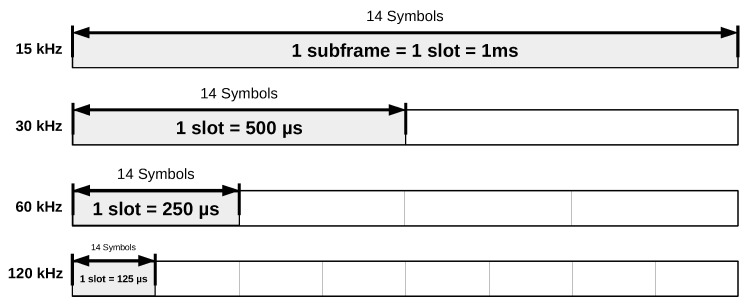
5G new radio (NR) frame structure.

The NR frame structure is the pivotal element for the flexible support of heterogeneous services, while allowing adaptation to different user channel conditions [6]. Flexibility here comprises different orthogonal frequency division multiplexing (OFDM)-based waveforms, as well as a mixed numerology [7]. The last term refers to different multicarrier modulation parameters with an impact on subcarrier spacing, cyclic prefix (CP) duration, and slot duration, as shown in Table 1. The downlink and uplink transmissions are organized into 10ms frames, each having ten subframes of 1ms. In 5G, the 1ms subframe is then divided into one or more slots, depending on the numerology index in use. As shown in Figure 1, the shortening of the slot duration is related to the shortening of the duration of the symbols.

On the one hand, this flexible design enables an efficient delivery of different qualities like low latency, guaranteed bit rate (GBR), reliability, and more [8]. On the other hand, 5G resource management becomes more challenging. Mixed numerologies are prone to: (i) inter-numerology interference (INI); (ii) low spectral efficiency; (iii) signaling overhead; (iv) scheduling complexity. Therefore, the number of coexisting numerology indexes should be minimized.

A mixed numerology system where different BWPs co-exist demands special planning. Such a planning problem involves deciding on the numerology mix and BWPs that better explore network capacity given a set of users/services that may change in number, requirements, and impairments. Such planning should also be feasible for environments having a dynamic traffic pattern. Here in this article, the pre-planning of the numerology profiles is proposed to address this issue. The mathematical optimization models created to design such mixed numerology profiles allow for the optimization of mixed numerologies in future 5G systems, under any wireless communication scenario and traffic pattern. More clearly, the contributions of this article are the following:

- A framework for the outline of mixed numerology profiles is proposed. These profiles are planned according to the *k* most antagonistic user/service requirements. The proposed framework allows a fast transition between profiles, performed in the case of traffic pattern changes.
- A two-step approach is proposed for the planning of such numerology profiles, each having its own mixed numerology and BWPs. Mathematical optimization models are presented to solve both steps.

As technology evolves in 5G, operators will be able to deliver even more advanced and value-added services. Therefore, network planning and deployments must be done in such a way that they match the ambition of the services. This requires understanding users/services and taking into account their requirements in order to outline future mixed numerology profiles. The proposed framework allows this goal to be reached.

The remainder of this article is organized as follows. Section 2 discusses related work, and then, Section 3 clarifies several wireless communication and 5G related definitions, which are required to understand the following sections and addressed problem. In Section 4, the motivation for the optimization of numerology profiles is presented, and a mathematical formulation of such an optimization problem is created, allowing it to be solved. Section 5 presents the simulation setup and analysis of the results, and Section 6 presents a final discussion on the results and conclusions, together with future work.

## 2. Related Work

The multiplexing of 5G services under a mixed numerology is now a hot topic. The mixture of OFDM-based numerology indexes was analyzed in [6], where it was shown to be beneficial for the support of different services with different latencies. However, due to energy leaks, the INI problem may appear. The authors demonstrated a time-domain window filtering method to reduce such an effect. The authors in [9] studied the multi-service support under different subcarrier spacing and highlighted the effect of the subcarrier spacing difference and the guard band size on the INI problem. They proposed a sub-band filtered transmission scheme and cancellation and equalization algorithms to reduce the effect of the INI problem. In [10], the INI problem was also analyzed and shown to be even more severe on the edge sub-carriers of neighboring BWPs. Similar to [9], the authors also showed that the effect of the INI is proportional to the difference in numerology indexes. In order to avoid the INI, an adaptive numerology selection approach was developed in [11] based on the delay requirements of the service. This approach considers only the delay requirements to reduce the average scheduling latency.

Finding the minimum mixture of numerology indexes was studied in [12], where a metric was developed to measure user satisfaction regarding the numerology assigned to deliver its services. Different scenarios were simulated and different numerology index sets considered. In order to select the desired number of numerologies, the authors proposed a greedy algorithm, which uses the metric to obtain a trade-off between scenario and user requirements. In [13], service multiplexing using a predefined mixture of numerology indexes was modeled as an integer programming problem and shown to be NP-hard by constructing a polynomial-time reduction from the partition problem. For this reason, it was solved using a Lagrangian relaxation. However, this work did not include the INI problem in the model. Similar studies can be found in [14,15]. In [14], a multi-user OFDM system was modeled using an integer program, and a relaxed version was developed to reduce the computational complexity. In [15], an integer program was also developed to model the scheduling of a multi-user system while meeting users’ service requirements. This work also proved that the multi-user resource allocation under different service requirements is an NP-hard problem. Moreover, the authors developed two different algorithms: a resource portioning algorithm to decompose the allocation problem into a set of parallel small scale problems and an iterative greedy algorithm based on the resource assignment weight. This work, however, allocated physical resource blocks (PRBs) to users under the assumption that numerologies are preselected by each user. The energy efficiency optimization problem was studied in [16]. Furthermore, a joint beamforming and power allocation scheme was proposed, which takes into consideration the intra- and inter-cell interference, but not the INI problem. In addition, resource scheduling of different services was studied in [17], where different models were developed to join enhanced mobile broadband (eMBB) and ultra reliable low latency communications (URLLC) resource allocation. In [18], the concern was to predict the outage probability in order to ensure efficient and stable service communications. New approaches using machine learning have also emerged, as in [19]. Table 2 summarizes the differences among these research efforts.

The previously mentioned studies considered either a predefined mixed numerology and addressed resource allocation and service multiplexing problems or considered planning a mixed numerology that is more suitable for specific services. In [20], we addressed the spectrum allocation problem. In this article, and contrary to such studies, the problem is to plan multiple numerological profiles that fit certain QoS requirements, which should be selected according to the presence or not of one or more of these QoS requirements over time. To our knowledge, this problem has not been addressed before.

## 3. Required Definitions

For readability, the notation used throughout the article is summarized in Table 3.

For now, 5G systems will use a single waveform (CP-OFDM), and two large frequency ranges (FRs) are specified by 3GPP: sub-6 GHz (FR1) and millimeter wave (FR2). A subcarrier spacing of 15 and 30 kHz can be used in sub-6 GHz; a subcarrier spacing of 120 kHz can be used in the millimeter wave range; and a subcarrier spacing of 60 kHz can be used in both. A set of bands has been defined for each FR, by 3GPP, together with the available subcarrier spacings and supported UE channel bandwidths (maximum transmission bandwidth + guard bands). For example, a 5 MHz UE channel bandwidth is only supported in a 15 kHz subcarrier spacing [23].

**Definition** **1** (Cell bandwidth). *Let us assume a wireless communication scenario in which the service area is covered by a specific frequency band B, under a certain bandwidth denoted by W. Guard band penalties are assumed to be incorporated in W, and the set of allowed numerology indexes is denoted by G(B,W).*

The cell bandwidth is expected to be large, but the reception/transmission bandwidth of a UE is not necessarily the same as that of the cell bandwidth. That is, the reception/transmission bandwidth of a UE will be a subset of the total cell bandwidth and may decrease during low activity to save power. A UE can have at most four BWPs configured for downlink (similar for uplink; supplementary uplink is possible), and for now, just a single BWP will be active at a time (in Release 15). One of the BWPs will be similar for all users in the cell and used for initial access to the network.

**Definition** **2** (BWP). *A bandwidth part is the frequency spectrum, within the carrier’s bandwidth, over which a device is currently operating. A BWP is a group of contiguous physical resource blocks (PRBs) where one PRB occupies 12 consecutive subcarriers (frequency domain), and it can be used in either direction (uplink or downlink). A BWP is associated with a numerology index, and only a single BWP can be active at any time. The set of BWPs for utilization by UE u is denoted by $P_{u}$, and if perc(p) is the bandwidth percentage assigned to $p\in P_{u}$, 0≤perc(p)≤1, then $\left\lfloor \frac{perc(p)\times W}{0.18\times 2^{g}}\right\rfloor$ will be the number of its PRBs (0.18MHz=15kHz×12) if numerology index g∈G(B,W) is used. A switch to short BWP allows energy saving, while BWPs at different numerology indexes allow for different services.*

The control mechanism responsible for exchanging BWP information is the radio resource control (RRC) protocol. This protocol is understood by both the user NR and by the network gNB. The RRC can perform BWP reconfiguration or use downlink control information (DCI) messages for BWP switching. These consume a certain time, and for this reason, their use should be minimized. Since the RRC reconfiguration is the most demanding one, the assigned BWPs should fit not only current user/service needs (while being efficient regarding the use of physical resources), but also future needs so that RRC reconfigurations are avoided as much as possible.

**Definition** **3** (NR data rate). *A user NR device is assumed to have a limitation on the speed at which it can transfer data, denoted by $C_{u}$. It is assumed that such a limitation is per 66.67 μs (symbol duration in Numerology Index 0). Such a limitation influences the user BWP sizes, numerology, and modulation scheme. That is, for a given BWP of user u, $\#PRBs\times 12\times m\times \left(g+1\right)\leq C_{u}$, for numerology g and modulation m.*

Moreover, NR devices also have a limitation on the speed at which they can transfer data, and impairments (e.g., Doppler spread, frequency offset) may exist.

**Definition** **4** (Impairments). *Measurements/feedback are provided via channel quality information (CQI), or other similar systems. The overall set of feedback elements is denoted by I, and it is assumed that specific wireless communication scenarios (e.g., mobile), served by a specific frequency band, usually lead to a set of channel and UE impairments. Such information should be used to improve the QoS.*

ITU-Rstarted by defining three service types: enhanced mobile broadband (eMBB), ultra reliable low latency communications (URLLC), and massive machine type communications (mMTC) [24]. Regarding these service types, it can be stated that:

- eMBB: The focus is on supporting the ever-increasing end user data rate and system capacity.
- URLLC: The focus is reliability and security required by mission-critical applications. High subcarrier spacing and mini-slots are the key enablers for this use case.
- mMTC: The focus is energy efficiency and massive connectivity.

This classification has as a basis the technical viewpoint of network operators and service providers. More recently, an end user experience perspective was proposed to classify 5G services [25,26]. The authors proposed a classification based on five features: immersiveness, intelligence, omnipresence, autonomy, and publicness. Naturally, other kinds of services may arise, allowing the possibility to better serve existing and future applications. As the number of classes increases, so does the complexity of systems because services will have different kinds of requirements.

The service requirements are specified through 5G QoS class index (5QI) values [27,28], and these are expected to differ among wireless communication scenarios like indoor hotspot, dense urban, rural, urban macro, high speed, etc. [29]. That is, wireless communication scenarios are expected to affect the service requirements, which is basically related to the impairments.

**Definition** **5** (Services). *The main types of uses that 5G is expected to enable (overall set of services under consideration) are denoted by S. The overall set of possible QoS requirements is denoted by Q, and it is assumed that requirement values change according to the wireless communication scenario.*

Regarding the duration of TTIs, these will change according to the used numerology index because TTI = the number of symbols in time × symbol length. For services requiring lower latencies, a low number of symbols per TTI or a short symbol length can be used to obtain a shorter TTI. For higher spectral efficiency, a longer TTI allows for higher spectral efficiency (less % of downlink control channel overhead, used to carry scheduling decisions every TTI). Therefore, there is a tradeoff between system spectral efficiency and minimal latency.

In practice, the subcarrier spacing, TTI, and number of subcarriers are directly related. For a low number of subcarriers, large subcarrier spacing is used, and a lower TTI (due to short symbol duration) is obtained, leading to low latency (note, however, that TTI has to do also with the number of symbols in time). Users/services can be flexibly multiplexed over the available resources with different TTIs, which allows for the support of service-aware TTI multiplexing on the same frequency [30]. The TTI can be adjusted according to the required latency and scheduling frequency [31].

The radio medium access control (MAC) scheduler does the packet treatment separately for each data radio bearer (DRB) following a two-step mapping of end-to-end (E2E) session flows.

**Figure 2:**
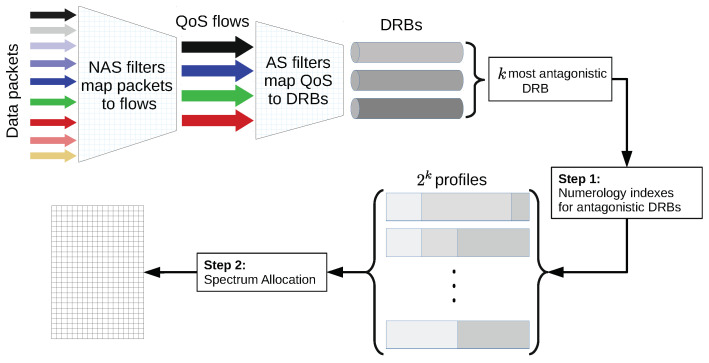
The two-step solution of the numerology selection problem. NAS, non-access stratum.

**Definition** **6** (Two-step mapping of E2E session flows). *The non-access stratum (NAS) filters the data packets in the UE, or 5G core network (CN), and associates the data packets with QoS flows. An E2E session can be associated with one or more QoS flows. The access stratum (AS) mapping in the UE (or 5G RAN) associates the QoS flows with the DRBs (see Figure 2). This mapping is based on 5QI in the transport header of the packets and on the corresponding QoS parameters that are signaled via the CN interface when a session is established. One or more QoS flows can be mapped to a DRB, and a UE can have a set of DRBs, denoted by $D_{u}=\left\{d_{u}^{1},\ldots ,d_{u}^{|D_{u}|}\right\}$. A DRB d has a vector of QoS requirement values denoted by $r^{d}=\left[r^{d}\left(q\right)\right]$, where q∈{1,…,Q}.*

Table 4 shows some traffic types and corresponding requirements. If the system reaches congestion, then priorities can always be used (see [28] for details). This two-step approach allows differentiating application/service flows and the adaptation of the DRB requirements to guide the radio scheduler. That is, the QoE manager can adaptively monitor and adjust the mapping of QoS flows to DRBs (reflective QoS). By adjusting the mapping of QoS flows to a DRB, there is an adjustment of the latency budget, packet loss rate tolerance, and GBR with the DRB, and this can be used to guide the lower-layer scheduler (although this should be done rarely, and priority should be changed to better serve an application). DRB data can be mapped to one or more BWPs and, consequently, TTI sizes.

Applications can have different requirements, and achieving the highest data rates may not always be the main requirement. Power consumption can be a critical issue and must also be considered. For most devices, the maximum throughput scenario is the one leading to the highest energy efficiency because the energy consumed per transferred bit is minimum (there is a power baseline that if distributed by a large number of bits, then the power efficiency is higher). However, it cannot be taken for granted that the power consumption will linearly map to a data rate improvement, especially if the device uses the full bandwidth to transmit lower data rate traffic (high baseline). That is, if the device uses a large bandwidth to transmit low data rate traffic, then there is energy inefficiency. In summary:

- Uplink: Since power varies according to the distance, when transmit power is near its limit, then the only way to extend uplink coverage is to concentrate the same energy into fewer bits. If the data are not urgent, then the BWP should be small. For short distances, higher BWPs (fitting data rate) should be used for higher energy efficiency).
- Downlink: High throughputs can be provided by high bandwidth carriers and MIMO layers, but this requires high processing capacity to deal with data rates and high power at maximum throughput because the device has to actively monitor wideband control channel across a large bandwidth, even when no data are present. For this reason, the BWP should be large in the case of high throughput, but small in the case of low throughput.

The power baseline also changes with the channel/BWP/modulation, and this will change the required energy per bit. Switching between BWPs can be done to keep the baseline low, if the throughput reduces, but the switching overhead must also be considered. In summary, the BWP and the consequent power consumption must be considered based on the traffic profile: service type and their requirements.

**Definition** **7** (NR energy). *NR devices are assumed to have different hardware characteristics. In general, the higher the channel, BWP, and modulation of a channel, the higher the power baseline. This should follow service throughput needs for energy efficiency. Full spectrum and short TTI allow constrained devices to go to sleep mode. BWP planning should take the traffic profile into account.*

Numerology indexes are not associated with specific service classes because there will be different communication scenarios and UE characteristics. For this reason, seven numerology indexes have been defined, and the BWP/numerology index in use by a UE may change at every TTI. This should be decided according to: (i) the requirements of services/DRBs in a communication scenario; (ii) NR features (bandwidth, data rate, and energy limitations); and (iii) impairments.

In general, few numerological indexes should be used when there is a need for greater spectral efficiency. This is because a mixture of numerology indexes requires guard bands to avoid the INI. In some cases, such spectral efficiency is more important than having multiple numerology indexes, which provides more flexibility, but at the expense of scheduling complexity and signaling overhead, leading to a waste of bandwidth resulting from the use of multiple guard bands.

## 4. Management of Radio Resources

### 4.1. Motivation

As previously described, the reception/transmission bandwidth of a UE is expected to be smaller than the cell bandwidth. Therefore, different numerology indexes and BWP sizes will co-exist. That is, at a given time, there is a numerology mix and combination of active BWPs at the network. The problem is, therefore, to decide on the numerology mix and BWPs that better explore network capacity given a set of users that may change in number, requirements, and impairments. Here, in this article, the pre-planning of numerology profiles is proposed to address this problem. More specifically, the idea is to explore requirements and impairments usually present in a given wireless communication scenario to build numerology profiles and then adopt one of the profiles according to the current users/traffic pattern.

### 4.2. Defining the Numerology Profile Problem

To better understand what is meant by numerology profiles, some definitions need to be introduced.

**Definition** **8** (Critical QoS requirement). *Let $r^{d}=\left[r^{d}\left(q\right)\right]$ denote the vector of QoS requirements for a DRB d, ∀q∈Q, where Q is the set of QoS requirements. Each element in $r^{d}$ is the relative need for QoS requirement q that results from QoS flows mapped to DRB d. Let $e_{g}^{d}=\left[e_{g}^{d}\left(q\right)\right]$ denote a vector with the relative effect of numerology index g on q, ∀q∈Q. Then, the most critical QoS requirement for DRB d, under numerology index g, is given by:*

(1) $$\Delta \left(d,g\right)=\underset{q\in \{1,\ldots ,Q_{d}\}}{arg max}\left\{\Phi \left(d,g,q\right)\right\},$$

*where $\Phi \left(d,g,q\right)=r^{d}\left(q\right)-r^{d}\left(q\right)\times e_{g}^{d}\left(q\right)$ measures the impact of numerology g on requirement q, for DRB d, assuming $0\leq e_{g}^{d}\left(q\right)\leq 1$.*

The most critical QoS requirement changes, therefore, according to the numerology index under utilization. The relative effect of numerology index *g* on QoS requirement *q* can be extracted from Table 5, and multiple requirements may have to be simultaneously considered. This is detailed when discussing the simulation setup in Section 5. A specific numerology can also have an opposite effect on different DRBs. That is, there will be antagonistic DRB critical requirements present at the network.

**Definition** **9** (Antagonistic DRBs). *Let us assume a wireless communication scenario with a representative set of DRBs with QoS requirements, denoted by D. Assuming $D_{k}$ is the set of all size-k subsets of D, then the k most antagonistic DRB critical requirements result from:*

(2) $$\underset{D_{k}^{i}\in D_{k}}{arg max}\{\sum_{l\in \{1\ldots k\}}\max_{g\in G}\{\sum_{m\neq l}|\Phi \left(d,g,q\right)-\Phi \left(d^{\prime },g,q^{\prime }\right)\left|\}\right\}$$

*where $d=D_{k}^{i}\left[l\right]$, $d^{\prime }=D_{k}^{i}\left[m\right]$, q=Δ(d,g), and $q^{\prime }=\Delta \left(d^{\prime },g\right)$.*

Both $r^{d}$ and $e_{g}^{d}$ values, used in Δ(.), are normalized between zero and one. Having these definitions in mind, the numerology profile problem can be defined as follows.

**Definition** **10** (Numerology profile problem). *Given a wireless communication scenario, with a representative set of DRBs with QoS requirements, denoted by D, then $P=2^{k}$ numerology profiles must be planned according to the presence of one or more of the k most antagonistic DRB critical requirements (possible combination of present DRBs). Their presence, at any moment in time, will lead to the adoption of one of the profiles by the network. Then, for each profile, it is necessary to decide on: (i) the most suitable numerology indexes; (ii) the percentage of spectrum to be allocated to each numerology index. Such a decision should try to ensure an optimal use of resources while providing fairness among users/services.*

Thus, given a wireless communication scenario, the numerology profiles reflect the anticipation of possible competitions for the spectrum by the *k* most antagonistic DRBs. Such planning criteria are flexible, using all degrees of freedom to reach the best multiplexing, depending on the presence (or not) of the *k* most antagonistic DRB critical requirements. Note that a high *k* value leads to fine-grained profiles.

Regarding impairments, it is assumed that these are incorporated in $e_{g}^{d}$ because certain wireless communication scenarios are known to exhibit specific impairments. Note also that due to the previously mentioned two-step mapping of E2E session flows, the requirements of DRBs in each wireless communication scenario are not expected to change much. Therefore, such a kind of long-term planning can always be applied to any wireless communication scenario.

### 4.3. Solving the Numerology Profile Problem

According to Definition 10, the numerology profile problem can be solved using a two-step approach (Figure 2). First, the numerology indexes that better serve the *k* most antagonistic DRB critical requirements, in each profile, should be found. Then, spectrum must be allocated to such numerology indexes, taking resource optimization and fairness into account. These two steps are discussed next.

#### 4.3.1. Most Antagonistic DRBs

Let us assume the following given information for a wireless communication scenario:
GSet of numerology indexes that can be used in the wireless communication scenario’s frequency band, where *g* denotes a specific numerology index.DSet of representative DRBs, where *d* denotes a specific DRB.*k*Maximum number of competing DRBs (granularity of profiles).QSet of QoS requirements.$r^{d}$Vector with |Q| relative QoS needs of DRB *d*.$e_{g}^{d}$Vector with the effectiveness of numerology *g* to accomplish the QoS needs of DRB *d*.

The variables are:
$\lambda^{d}$One if DRB d∈D is considered one of the *k* most antagonistic DRB critical requirements; zero otherwise.$\Delta_{q,g}^{d}$One if q∈Q is the most critical QoS requirements when DRB d∈D is under numerology index g∈G; zero otherwise.$\gamma_{q,q^{\prime },g}^{d,d^{\prime }}$One if critical requirements *q* and $q^{\prime }$ of antagonistic DRB $\left(d,d^{\prime }\right)$ pair, under numerology *g*, is to be considered when evaluating the expression in Definition 10; zero otherwise.$\mu_{q,q^{\prime },g}^{d,d^{\prime }}$Difference between critical requirements *q* and $q^{\prime }$ of antagonistic DRB $\left(d,d^{\prime }\right)$ pair, under numerology *g* (|⋯| in Equation (2)).

Given such notation, this step can be solved by the following mathematical optimization problem:

Objective function:

(3) $$\mathrm{maximize}\sum_{\left\{d,d^{\prime }\in D\right\}}\sum_{\left\{q\in Q_{d}\right\}}\sum_{\left\{q^{\prime }\in Q_{d^{\prime }}\right\}}\sum_{\left\{g\in G\right\}}\mu_{q,q^{\prime },g}^{d,d^{\prime }}$$

A numerological index *g* will have different suitability degrees for each DRB QoS requirement. For fairness among users/services, the DRBs with the greatest difference in suitability (given a specific numerology index) should be the ones to take into account when choosing the numerology indexes in operation. This objective function searches for those opposite suitabilities, or most antagonistic DRBs.

Competing DRBs:

(4) $$\sum_{\left\{d\in D\right\}}\lambda^{d}=k$$

This constraint states that *k* DRBs, the most antagonistic, must be determined. This depends on the supported numerology index mixing level.

Critical QoS requirement:

(5) $$\sum_{\left\{q\in Q\right\}}\Delta_{q,g}^{d}=\lambda^{d},\forall d\in D,\forall g\in G$$

Constraints (5) are used to ensure that a single QoS requirement is marked as the critical one, when DRB *d* is operating in numerology number *g* and is one of the most antagonistic DRBs. The right QoS requirement, to be considered as critical, is influenced by the objective function maximization and the next constraints.

Most competing DRBs:

(6) $$\begin{array}{c} \gamma_{q,q^{\prime },g}^{d,d^{\prime }}\leq \Delta_{q,g}^{d},\forall d,d^{\prime }\in D,\forall q\in Q_{d},\forall q^{\prime }\in Q_{d^{\prime }},\forall g\in G \end{array}$$

(7) $$\begin{array}{c} \gamma_{q,q^{\prime },g}^{d,d^{\prime }}\leq \Delta_{q^{\prime },g}^{d^{\prime }},\forall d,d^{\prime }\in D,\forall q\in Q_{d},\forall q^{\prime }\in Q_{d^{\prime }},\forall g\in G \end{array}$$

(8) $$\begin{array}{c} \mu_{q,q^{\prime },g}^{d,d^{\prime }}=\gamma_{q,q^{\prime },g}^{d,d^{\prime }}\times \left|\left[r^{d}\left(q\right)-r^{d}\left(q\right)\times e_{g}^{d}\left(q\right)\right]-\left[r^{d^{\prime }}\left(q^{\prime }\right)-r^{d^{\prime }}\left(q^{\prime }\right)\times e_{g}^{d}\left(q^{\prime }\right)\right]\right|,\\ ,\forall d,d^{\prime }\in D,\forall q\in Q_{d},\forall q^{\prime }\in Q_{d^{\prime }},\forall g\in G \end{array}$$

Constraints (6) and (7) find $\left(d,d^{\prime }\right)$ pairs of antagonistic DRB critical requirements, those that must be considered when evaluating the expression in Definition 10. Their suitability difference, when using *g*, is stored in (8). Note that variables $\lambda^{d}$, $\Delta_{q,g}^{d}$, $\gamma_{q,q^{\prime },g}^{d,d^{\prime }}$, and $\mu_{q,q^{\prime },g}^{d,d^{\prime }}$ depend on each other, and their values are decided globally, as a whole, guided by the maximization goal as the objective function.

Non-negativity assignment to variables:

(9) $$\begin{array}{c} \lambda^{d},\Delta_{q,g}^{d},\gamma_{q,q^{\prime },g}^{d,d^{\prime }}\in \left\{0,1\right\};\mu_{q,q^{\prime },g}^{d,d^{\prime }}\geq 0. \end{array}$$

#### 4.3.2. Spectrum Allocation

After determining the most antagonistic DRBs, spectrum allocation must be performed while ensuring fairness among users/services. This requires introducing some assumptions based on the discussion in Section 4. These assumptions will serve as a basis for the optimization problem presented next.

**Assumption** **1** (Q1: Latency and GBR). *Services with latency and GBR limitations should have a short TTI, allowing a high scheduling frequency. Large BWPs (for less symbols in time) and/or high numerology indexes should be adopted.*

**Assumption** **2** (Q2: Data rate). *Services requiring high data rates should have a large channel bandwidth. A large TTI, for high spectral efficiency (less downlink scheduling control info), and a high modulation scheme should be used.*

**Assumption** **3** (Q3: Packet error rate). *For services requiring low packet loss, extended CP and low modulation schemes should be applied. The first acts as a guard band between successive symbols, overcoming inter-symbol interference (ISI) and enabling reliable operation.*

Now, let us assume the following known information:
$\bar{D}$Antagonistic DRBs determined in Step 1: $\bar{D}=\left\{d\in D:\lambda^{d}=1\right\}$.$r^{d}$Vector with relative QoS needs of DRB $d\in \bar{D}$.$e_{g}^{d}$Vector with the effectiveness of numerology *g* to accomplish the QoS needs of DRB *d*.*k*Maximum number of competing DRBs (granularity of profiles).PSet of spectrum uses/profiles, of size $2^{k}$, where *p* denotes one of the profiles.$a_{i}^{d}$One if DRB $d\in \bar{D}$ is associated with position i∈{1…k}; zero otherwise.$c_{p}^{i}$One if position i∈{1…k} is active in profile p∈P; zero otherwise.${\bar{G}}_{p}$Numerology indexes for profile *p* given by ${\bar{G}}_{p}=\left\{g:g=\underset{g\in G}{arg min}\left\{\max_{q\in Q_{d}}\left\{r^{d}\left(q\right)-r^{d}\left(q\right)\times e_{g}^{d}\left(q\right)\right\}\right\},\forall d:a_{i}^{d}\times c_{p}^{i}=1\right\}$.
Regarding ${\bar{G}}_{p}$, this will store the best numerology indexes for each $d\in \bar{D}$. More specifically, max gives the worst impact of numerology index *g*, from all QoS requirements *q* of DRB *d*, and arg min chooses the lowest value (numerology index with the least reduction of QoS). The variables are:
$\alpha_{p,g}^{L}$Lowest PRB for numerology index $g\in {\bar{G}}_{p}$ in profile p∈P.$\alpha_{p,g}^{H}$Highest PRB for numerology index $g\in {\bar{G}}_{p}$ in profile p∈P.$\Theta_{p}$Lower bound on the numerology index spectrum allocations in profile p∈P.

Given such notation, the spectrum allocation can be modeled by the following mathematical optimization problem:

Objective function:

(10) $$\mathrm{maximize}\sum_{\left\{p\in P\right\}}\Theta_{p}$$

In this second step, the objective is to maximize the lower bound on the numerology index spectrum allocation, for each profile, in order to ensure fairness among spectrum allocations.

Allocation of PRBs:

(11) $$\alpha_{p,g}^{L}\leq \alpha_{p,g}^{H},\forall p\in P,\forall g\in {\bar{G}}_{p}$$

(12) $$\sum_{\left\{g\in {\bar{G}}_{p}\right\}}\left(\alpha_{p,g}^{H}-\alpha_{p,g}^{L}\right)\times 2^{g}+[B\times (|{\bar{G}}_{p}\left|-1)\right]\leq W,\forall p\in P$$

where *B* and *W* are, respectively, the numerology guard bands and the total bandwidth in 0.18MHz (15kHz×12) PRB units. Constraints (11) arrange the lower and upper PRB limits of the numerology indexes included in profiles, while Constraints (12) avoid exceeding the overall bandwidth in every profile.

(13) $$\left(\alpha_{p,g}^{H}-\alpha_{p,g}^{L}\right)\times E_{g}\geq M_{p,g},\forall p\in P,\forall g\in {\bar{G}}_{p}$$

These constraints ensure a minimum bandwidth, $M_{p,g}$, required to ensure the packet delay budget for services with latency and GBR requirements (Assumption 1). The efficiency values, $E_{g}$, are given in Table 1.

Fairness inside profiles:

(14) $$\Theta_{p}\leq \left(\alpha_{p,g}^{H}-\alpha_{p,g}^{L}\right)\times 2^{g}\times E_{g}\times \frac{1}{R_{p,g}},\forall p\in P,\forall g\in {\bar{G}}_{p}$$

These constraints ensure that in each profile, the numerology indexes get fair BWPs. Note that the efficiency will be lower for numerology indexes with a large CP, so Assumption 3 is accounted for through the use of $E_{g}$, $0\leq E_{g}\leq 1$. $R_{p,g}$ is a relative data rate required in numerology index *g*, under profile *p*, allowing for spectrum allocation to be proportional to service needs in $r^{d}$ (considering all DRBs better served by numerology *g* in use at profile *p*). This accounts for Assumption 2.

Non-negativity assignment to variables:

(15) $$\alpha_{p,g}^{H},\alpha_{p,g}^{L},\Theta_{p}\in R^{+}.$$

The CPLEX (IBM ILOG CPLEX Optimizer Version 12.8.) optimizer is used to optimally solve both steps of the numerology profile selection problem. Note that the first step of the numerology profile problem will be solved sporadically, when DRB requirements change significantly (which happens rarely, as stated in Section 3), and can always be solved offline. The second step would be solved when DRB data rates change significantly, but the developed mathematical formulation is a linear programming (LP) problem and can be solved in polynomial time, which means that it will be solved quickly even for large instances [32]. It can also be solved offline. In the other circumstances, the operators should switch between pre-planned profiles according to the currently present DRBs.

### 4.4. User BWP Assignment

Under each profile in operation, the user BWP allocation should be decided in real-time so that UE characteristics are taken into account, which can be ensured by the scheduler. Such allocation must not only take Assumptions 1–3 into account, but also the device energy constraints, as follows:

**Assumption** **4** (Energy). *For energy-constrained devices, the channel bandwidths will be low, and under normal conditions, the BWP/modulation should fit the expected throughput for energy efficiency. A short TTI (high numerology index) allows the device to enter sleep mode. When using high power (distance related) for transmission, the BWP/modulation should be small.*

## 5. Analysis of the Results

### 5.1. Relative Effect of Numerology Indexes

There are different supported scheduling approaches: slot based and non-slot based [33]. Moreover, the number of OFDM symbols per TTI can be different (Table 1). In other words, resources can be scheduled for users on a complete slot basis (14 OFDM symbols), or mini-slot scheduling can be used. A mini-slot is typically 2, 4, or 7 symbols [34]. A short TTI can be obtained by increasing the subcarrier spacing, Δf, or reducing the number of symbols per TTI. With this in mind and considering the information in Table 5, the relative effect of numerology indexes, $e_{g}^{d}$, is determined for the services in Table 4 as follows:

- VoIP, video conferencing, and real-time gaming: For these services, a GBR is required. Therefore, a short TTI duration is necessary. For video conferencing and real-time gaming, higher numerology indexes should be used due to their relatively high data rate requirements. Moreover, for real-time gaming, high numerologies can ensure the required low latencies. For VoIP, mini-slots in low numerologies can be used.
- Buffered streaming: In this type of application, the latency requirements are not strict. Therefore, a large TTI can be adopted. Whenever packet retransmission is need, packet prioritization and/or buffering techniques can be used to cope with the required low packet error rate. Therefore, low numerologies should be used.
- Live streaming and pre-AR/VR: Since pre-AR/VR requires firm latency requirements and low packet error rates, Numerology Index 2 using extended CP can be used. Live streaming requires a smaller packet error rate, has no GBR requirements, and has some latency concerns. Therefore, middle numerology indexes (with normal CP) can be used.

The relative effect of numerology indexes for services in Table 4 is summarized in Table 6.

### 5.2. Simulation Set Up

Let us assume an overall bandwidth *W* = 50 MHz, which is supported in multiple numerologies, corresponding to a total number of 270 PRBs in 0.18 MHz (15 kHz × 12) PRB units [23,35]. For simplicity, let us assume a fixed guard band of *B* = 50 kHz between any two numerologies, which is ≈ 0.28 in 0.18 MHz PRB units [12].

For services with latency and GBR requirements, the minimum bandwidth to ensure the packet delay budget, $M_{p,g}$, can be obtained as the following: Let $T_{\mathrm{symbol}}=\frac{T_{\mathrm{slot}}}{14}$ be the symbol duration, where $T_{\mathrm{slot}}$ is obtained from Table 1; the maximum number of allowed symbols in time, due to latency constraints, will be $\frac{L}{T_{\mathrm{slot}}/14}$, and $M_{p,g}$ will be $\frac{GBR}{\frac{L}{T_{\mathrm{slot}}/14}\times m}$ subcarriers, where *m*, GBR, and *L* are the maximum modulation factor, the total GBR needs of DRBs fitting numerology index *g*, and the average latency requirement of these DRBs, respectively. For the $E_{g}$ values, these are shown in Table 1. Therefore:

$$M_{p,g}=\frac{GBR\times T_{\mathrm{slot}}}{L\times 14\times m\times 12}$$

in PRB units. Note that $T_{\mathrm{slot}}$ is dependent on the numerology. Regarding $R_{p,g}$, it can be obtained from the data rates shown in Table 4. The just mentioned simulation parameter values are summarized in Table 7.

### 5.3. Discussion

#### 5.3.1. Antagonistic DRBs and Numerologies

Table 8 and Table 9 show the set of antagonistic DRBs and numerology indexes that better serve these antagonistic DRBs according to Table 6, for different profile granularities (maximum number of competing DRBs considered, *k*). The information in these tables basically includes the outcome of Step 1, from the two-step approach adopted to solve the numerology profile problem. Note that a high *k* value leads to fine-grained profiles.

According to these results, the most antagonistic DRBs are video conferencing and Pre-AR/VR, and then VoIP, buffered streaming, real-time gaming, and live streaming as the granularity of the profiles, *k*, increases. The numerology indexes that best meet the requirements of such applications are Numerology Index 2 with extended CP (2eCP) and 4 for k=2. For k=3, the best numerology indexes are 0, 2eCP, and 4. These three numerology indexes seem to be adequate for all applications except live streaming, and that is why no extra numerology index is selected until the live streaming is included as an antagonistic DRB, for which Numerology Index 2 is more adequate. Therefore, not all numerology indexes will be used, even if all applications are assigned to their best numerology index. Of course, this depends on the wireless communication scenario and kind of DRBs/applications under consideration. The numerology index that best meets the requirements of each application is shown in Table 10.

#### 5.3.2. Spectrum Allocation

**Figure 3:**
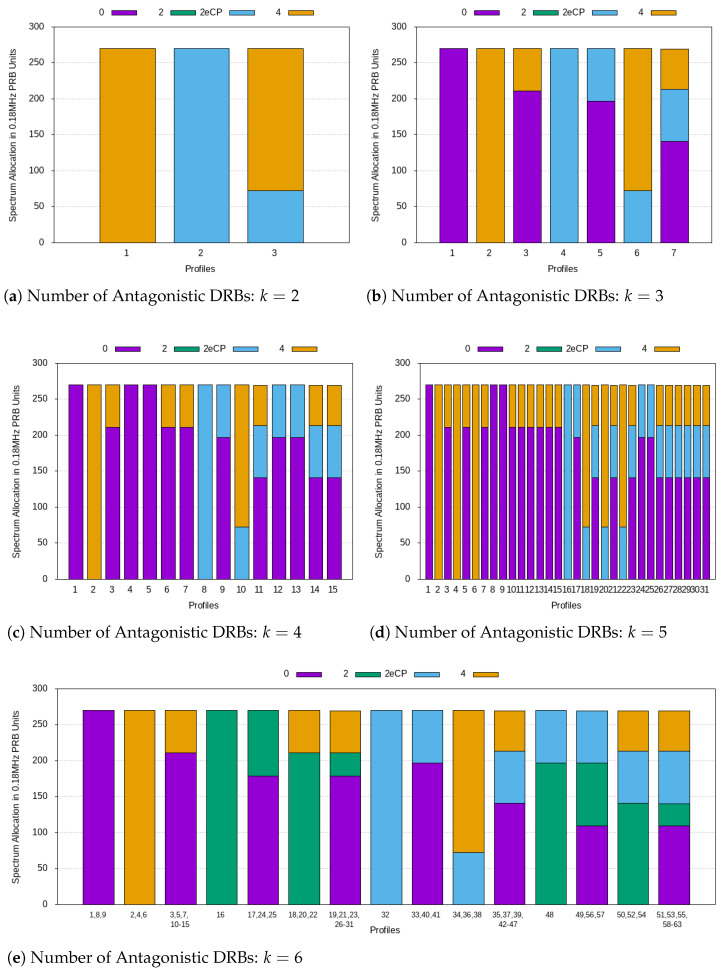
Spectrum allocation.

The plots in Figure 3 show the spectrum allocation in 0.18MHz (15kHz × 12) PRB units. For k=4 and k=5, the set of numerology indexes in operation will be the same as for k=3. In other words, although the set of possible profiles increases, some of them activate the same numerology indexes. For this reason, these are grouped when plotting spectrum allocation for k=6.

Each of the $P=2^{k}$ numerology profiles being planned can be represented by a *k*-bit pattern. An activated bit corresponds to the presence of an antagonistic DRB in the wireless communication scenario and is to be considered when distributing spectrum. Such a design of numerology profiles has the following advantages:

- Changes in users/services, currently present in a wireless communication scenario, can be followed by non-abrupt changes in spectrum allocation. That is, multiple transitions between numerology profiles, differing in a single bit, can be performed until the final numerology profile is reached. This allows for spectrum reconfiguration to be gradually introduced, avoiding posing problems to the users/services.
- When moving from one profile to another, at most one currently operating numerology index will have to be redefined to insert another numerology index.

These features allows for RRC reconfigurations (required for the new spectrum distribution to take place) to be kept minimized while ensuring full fairness of spectrum allocation among users/services.

#### 5.3.3. Overall Spectral Efficiency

**Figure 4:**
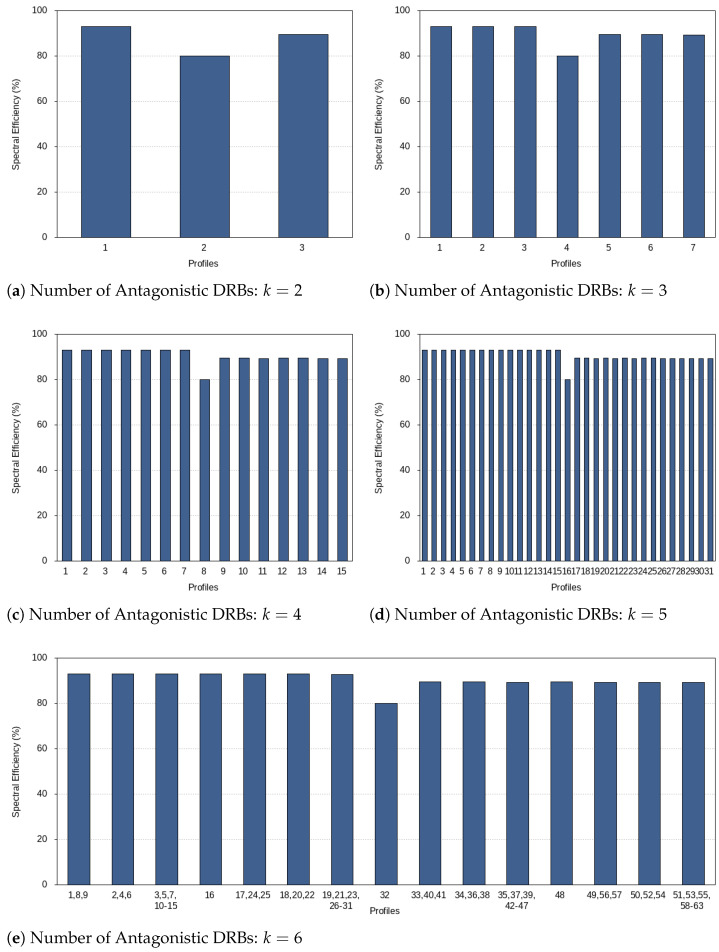
Overall spectral efficiency.

The plots in Figure 4 show the spectral efficiency in the determined profiles. In general, the greater the amount of spectrum allocated to numerology index 2eCP, the lower the spectral efficiency is, as expected. Please note that these plots refer to the overall spectral efficiency, and fair spectrum allocation among the numerology indexes is guaranteed. This is ensured by the expression (14) included in the mathematical optimization solving the second step of the numerology profile problem. Fairness is proportional to the relative data rate need in operating numerology indexes. This ensures equal quality of experience for the different users/services.

#### 5.3.4. Satisfaction of QoS Requirements

**Figure 5:**
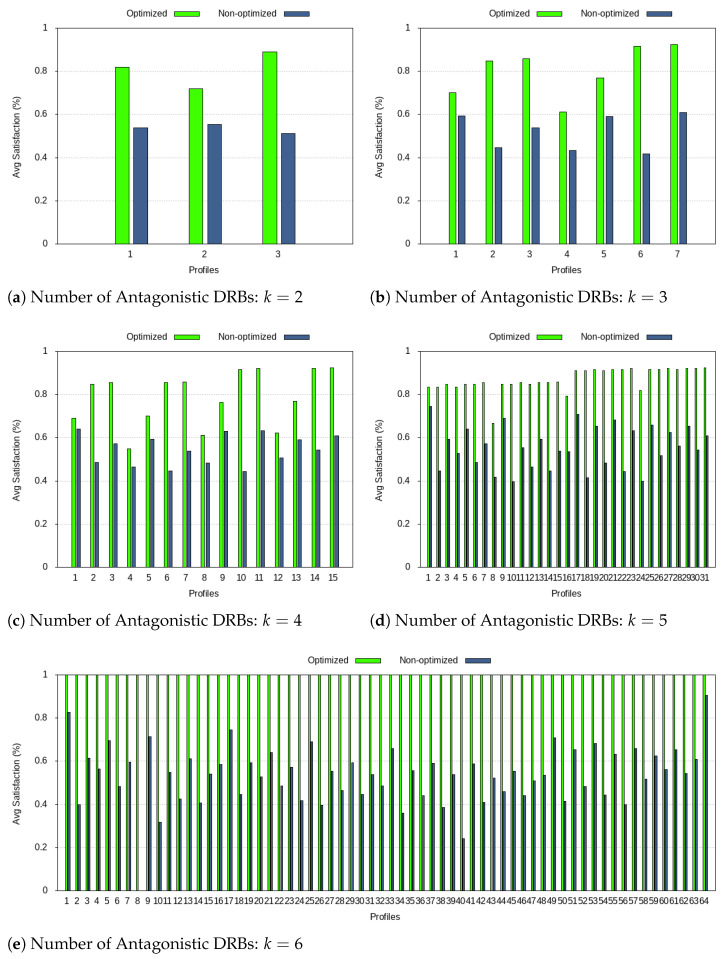
Average satisfaction of QoS requirements.

The plots in Figure 5 show the average satisfaction of user/application requirements. The “optimized” bar is obtained by applying $\Phi \left(d,g,q\right)=r^{d}\left(q\right)-r^{d}\left(q\right)\times e_{g}^{d}\left(q\right)$ to the numerology index that best fits DRB *d*, from the numerology indexes available in a certain profile, and averaging the values obtained for all requirements, $q\in \{1,\cdots ,Q_{d}\}$. That is, according to the traffic types under consideration, shown in Table 4, *q* will be latency, average data rate (DR), GBR, and PER. The “non-optimized” bar is obtained by taking the least suited numerology index. Note that the average satisfaction goes from 0% to 100% because $0\leq e_{g}^{d}\left(q\right),r^{d}\left(q\right)\leq 1$.

Results show that the optimization approach is able to choose adequate numerology indexes. The ranges for the “optimized” and “non-optimized” satisfaction percentage values are [0.6–1] and [0.4–0.65], respectively, and the values improve as the number of antagonistic DRBs considered, *k*, increases. Depending on the profile, the satisfaction level of the “optimized” approach can be twice as much as the “non-optimized” one. Therefore, the mathematical optimization is able to find numerology indexes that better meet user/service requirements, while keeping the total number of numerology indexes in operation minimized. For k=6, one-hundred percent satisfaction is reached because the mixed numerology includes all required numerology indexes, fitting all requirements adequately.

### 5.4. Applicability of the Model

According to TS38.321, the RRC method is used for the configuration of a BWP set at any stage of the call, but the processing of RRC messages can reach 10 ms. On the other hand, the DCI-based adaptation allows on-the-fly activation/switching of a BWP, and the latency can be as low as 2 ms. Since RRC reconfigurations are the most demanding ones, the initially configured BWPs should fit not only current user/service needs (while being efficient regarding the use of physical resources), but also future needs, for an on-the-fly activation of BWPs. Besides BWPs, the network also informs a UE of the cell bandwidth.

The proposed framework is useful for the planning of BWPs on the network side. The use of profiles helps define adequate BWP sets, to be configured in a UE at the beginning of calls. This promotes on-the-fly activation of BWPs during the call, instead of RRC reconfigurations. The proposed framework can be applied to any wireless communication scenarios because the relative effect of each numerology index, on each QoS requirement, is set individually, and these can be adjusted according to the impairments of the scenario. Since the requirements of DRBs, typically present in the communication scenario, are not expected to change much, such planning of long-term profiles is feasible.

## 6. Conclusions and Future Work

This article addresses the mathematical optimization of numerology profiles. These numerology profiles reflect the anticipation of possible competitions for spectrum among the most antagonistic DRBs. The developed mathematical model is flexible, using all degrees of freedom to reach the best multiplexing, depending on the presence (or not) of the DRBs. The results show that the numerology indexes under utilization and spectrum allocation are fully optimized, according to users/service needs, while minimizing the number of reconfigurations required for the accommodation of new DRBs, avoiding disrupting other users/services. Furthermore, depending on the granularity of the profiles, the proposed optimization model is able to reach satisfaction levels of 60–100%, whereas a non-optimized approach reaches 40–65%, while simultaneously minimizing the total number of numerology indexes in operation. The future work will focus on developing a machine learning solution for profile selection and transition. Since profile selection can be converted into a sequential decision problem, we will develop an adaptive reinforcement learning solution to this problem and study it under different use cases and deployment scenarios.

## Figures and Tables

**Table 1:** Numerology structure in 5G.

Index	Δf (kHz)	$T_{\mathrm{CP}}$ (μs)	$T_{\mathrm{slot}}$ (ms)	Efficiency
0	15	4.7	1	93.4%
1	30	2.3	0.5	93.4%
2	60	1.2/4.2	0.25	93.4/80.0%
3	120	0.6	0.125	93.4%
4	240	0.3	0.0625	93.4%

**Table 2:** Mixed numerology related work.

Ref.	Year	Main Focus	Outcome	Pros and Cons
[6]	2016	Analyzing the mixture of numerologies to support different services.	Showed that multiple numerologies are beneficial for supporting different services and proposed a time-domain window filtering (W-OFDM) method to reduce the effect of the inter-numerology interference (INI).	W-OFDM suffers from imperfect power amplifier (PA) nonlinearity.
[9]	2017	Multi-service support under different subcarrier spacing.	Highlighted the effect of the subcarrier spacing difference and the guard band size on the severity of the INI problem and proposed a sub-band filtered transmission scheme using cancellation and equalization algorithms to reduce the effect of the INI problem.	Accurate channel state and noise variance feedback are required for proper cancellation [21].
[10]	2018	The INI problem effect across the sub-carriers.	Showed that the INI is more severe on the edge sub-carriers of neighboring bandwidth parts (BWPs).	Only simulation results under systems with adjacent bands were analyzed.
[16]	2018	Energy efficiency optimization.	A scheme that joins beamforming and power allocation.	Considered the intra- and inter-cell interference, but not the INI problem.
[12]	2018	The minimum mixture of numerologies for a scenario.	A metric is to measure user satisfaction, and a greedy algorithm to obtain a trade-off between scenario and user requirements.	A simple metric and algorithm, but not optimal.
[13]	2018	Modeling the service multiplexing using a predefined mixture of numerology indexes.	An integer program, and a Lagrangian relaxation solution.	Did not consider the INI in the model.
[11]	2019	Adaptive numerology selection.	An adaptive selection approach able to reduce the average scheduling latency.	Considered only the delay requirements.
[14]	2019	Modeling the multi-user OFDM system under a mixture of numerologies.	An integer program and a relaxed solution.	Assumed a fixed mixture of numerologies.
[20]	2020	Resource allocation under different services and a mixture of numerologies.	An optimization model (a linear program).	Can solve the problem in polynomial time, but did not include profile planning.
[15]	2020	Modeled the scheduling of a multi-user system under different service requirements.	An integer program, a resource partitioning algorithm, and an iterative greedy algorithm.	Allocated physical resource blocks (PRBs) to users under the assumption that numerologies are preselected by each user.
[17]	2020	Modeled the joint enhanced mobile broadband (eMBB) and ultra reliable low latency communications (URLLC) scheduling and analyze the eMBB rate loss associated with URLLC superposition.	Different loss function models: linear, convex, and threshold; and a solution for each model.	The accuracy of the linear model was not high in practice [22].
[19]	2020	Resource allocation under different numerologies.	A deep reinforcement learning solution for resource allocation.	Did not consider a mixture of numerologies.

**Table 3:** Notation. CQI, channel quality information; DRB, data radio bearer.

Symbol	Meaning
*B*	Frequency Band Covering the Service Area.
*W*	Maximum transmission bandwidth covering the service area.
G(B,W)	Set of allowed numerology indexes for frequency band *B* under bandwidth *W*; *g* denotes an element of this set.
$P_{u}$	Set of BWPs for utilization by UE *u*; *p* denotes an element of this set.
perc(p)	Bandwidth percentage assigned with BWP *p*.
$C_{u}$	Transfer data limitation for UE *u*, per 66.67us(symbol duration in Numerology Index 0).
*m*	Modulation data transfer factor.
I	Set of measurements/feedback provided via CQI or other similar systems.
S	Set of services.
Q	Set of QoS requirements; *q* denotes an element of this set.
D	Set of DRBs in a wireless communication scenario.
$D_{u}$	Set of DRBs for UE *u*; *d* denotes an element of this set.
$r^{d}$	Vector of QoS requirement values for DRB *d*; $r^{d}\left(q\right)$ denotes the value of requirement *q*.
$e_{g}^{d}$	Vector with the relative effect of numerology index *g* on QoS requirements, for DRB *d*; $e_{g}^{d}\left(q\right)$ denotes the effect on requirement *q*, $0\leq e_{g}^{d}\left(q\right)\leq 1$.
Δ(d,g)	The most critical QoS requirement, for DRB *d*, under numerology index *g*.
Φ(d,g,q)	Impact of numerology *g* on QoS requirement *q*, for DRB *d*.
$D_{k}$	Set of all size-*k* subsets of D; $D_{k}^{i}$ denotes one of the subsets, and $D_{k}^{i}\left[l\right]$ denotes a DBR in subset $D_{k}^{i}$.
$\bar{D}$	Most antagonistic DRBs.
*k*	Maximum number of competing DRBs to be considered.
P	Set of numerology profiles, of size $2^{k}$; *p* denotes one of the profiles.
*P*	Number of numerology profiles.
${\bar{G}}_{p}$	Indexes used in numerology profile *p*.

**Table 4:** Requirements for traffic types. 5QI, 5G QoS class index.

Application	Latency	Avg Data Rate	Guaranteed Bit Rate	Packet Error Rate	5QI Value
	(ms)	(Mb/s)	(Mb/s)		
VoIP	100	0.16	0.112	$10^{-2}$	1
Video conference	150	1	0.8	$10^{-3}$	2
Real-time gaming	50	0.8	0.72	$10^{-3}$	3
Buffered streaming	300	3.33	-	$10^{-6}$	6
Live streaming	100	1	-	$10^{-3}$	7
Pre-AR/VR	10	2	-	$10^{-6}$	80

**Table 5:** Numerology design needs for service requirements. TTI, transmission time interval; GBR, guaranteed bit rate.

Requirement	Δf	# of Subcarriers	TTI	$T_{\mathrm{CP}}$	Spectral	Comment
	(kHz)		Duration	(μs)	Efficiency	
High data rate					High	Less scheduling control info
GBR			Short			High scheduling frequency
Latency	Large		Short			Short symbol duration
Low packet error	Large			Long		Low inter-symbol interference
Energy efficiency		Low	Short			Low baseline and sleep mode
						increase

**Table 6:** Effect of numerology indexes for services in Table 4.

Service	g=0	g=1	g=2	g=3	g=4
VoIP	1 *	0.75	0.5	0.25	0
(if *q* is L/DR/GBR)					
Video conference	0	0.25	0.5	0.75	1
(if *q* is L/DR/GBR)					
Real-time gaming	0	0.25	0.5	0.75	1
(if *q* is L/DR/GBR)					
Buffered streaming	1	0.75	0.5	0.25	0
(if *q* is L/GBR)					
Live streaming	0	0.5	1	0.5	0
(if *q* is L/PER)					
Pre-AR/VR	0	0	1 **	0	0
(if *q* is L/PER)					

**Table 7:** Simulation parameters.

Parameter	Value
Bandwidth, *W*	50 MHz
Guard band, *B*	50 kHz
Modulation order	1024
Available numerology indexes	0–4; see Table 1
DRB services	see Table 4
DRB’s GBR and latency requirements	see Table 4

**Table 8:** Most antagonistic DRBs.

Application	k=2	k=3	k=4	k=5	k=6
VoIP	-	✓	✓	✓	✓
Video conference	✓	✓	✓	✓	✓
Real-time gaming	-	-	-	✓	✓
Buffered streaming	-	-	✓	✓	✓
Live streaming	-	-	-	-	✓
Pre-AR/VR	✓	✓	✓	✓	✓

**Table 9:** Selected numerologies.

Numerology Index	k=2	k=3	k=4	k=5	k=6
0	-	✓	✓	✓	✓
1	-	-	-	-	-
2	-	-	-	-	✓
2eCP *	✓	✓	✓	✓	✓
3	-	-	-	-	-
4	✓	✓	✓	✓	✓

2eCP * = Numerology Index 2 with extended CP.

**Table 10:** Numerology indexes that best meet the application requirements.

Application	k=2	k=3	k=4	k=5	k=6
VoIP	4	0	0	0	0
Video conference	4	4	4	4	4
Real-time gaming	4	4	4	4	4
Buffered streaming	4	0	0	0	0
Live streaming	4	0	0	0	2
Pre-AR/VR	2eCP	2eCP	2eCP	2eCP	2eCP

## Data Availability

Data sharing not applicable.
